# Five-year real-world outcomes of short-course leukocyte-poor PRP versus standard conservative therapy in early-stage knee osteoarthritis

**DOI:** 10.1371/journal.pone.0344749

**Published:** 2026-04-02

**Authors:** Qiuping Yu, Chenghong Wen, Junli Liu

**Affiliations:** 1 Health Management Center, West China Hospital/West China School of Medicine, Sichuan University, Chengdu, Sichuan, People’s Republic of China; 2 Affiliated Sport Hospital of Chengdu Sport University, Chengdu, Sichuan, People’s Republic of China; 3 Department of Orthopedics, Chongqing General Hospital, Chongqing University, Chongqing, People’s Republic of China; The Affiliated Changzhou No 2 People's Hospital of Nanjing Medical University, CHINA

## Abstract

**Background:**

Platelet-rich plasma (PRP) is increasingly applied in knee osteoarthritis (KOA), but its long-term efficacy remains uncertain. This study assessed 5-year outcomes of intra-articular PRP compared with conventional therapy in early-stage KOA.

**Methods:**

A retrospective cohort of 116 patients with bilateral KOA (Kellgren–Lawrence I–II) treated in 2019 was analyzed. Group A (n = 58) received standardized management plus three PRP injections, while Group B (n = 58) received standardized conservative management alone (NSAIDs and/or intra-articular hyaluronic acid), combined with education, exercise therapy, and weight management. Pain, function, and quality of life were evaluated by VAS, WOMAC, and SF-36 at baseline and 5 years.

**Results:**

Both groups showed deterioration over 5 years. VAS increased from 4.64 ± 0.52 to 5.41 ± 1.17 in Group A and from 4.80 ± 0.59 to 5.44 ± 1.16 in Group B, without significant between-group difference (*P* = 0.849). WOMAC and SF-36 scores also worsened similarly, with no intergroup significance (*P* > 0.05). Intra-group analyses confirmed significant declines from baseline (*P* < 0.001).

**Conclusions:**

Although a short, single course of leukocyte-poor PRP did not yield sustained superiority over standardized conservative management at 5 years, this real-world study provides rare long-term comparative evidence that defines the temporal boundaries of PRP efficacy and underscores the need for maintenance or optimized dosing protocols in clinical practice.

## Introduction

Knee osteoarthritis (KOA) is a common, progressive degenerative joint disease involving the entire joint as an organ. It is characterized by cartilage degradation, subchondral bone remodeling, synovial inflammation, meniscal degeneration, and pathological changes in the infrapatellar fat pad, resulting from complex interactions among mechanical stress, inflammation, and metabolic imbalance [1]. Inflammatory mediators such as interleukin-1β and tumor necrosis factor-α promote matrix metalloproteinase activation and cartilage breakdown. Globally, KOA affects over 250 million individuals, with prevalence rising due to aging and obesity [2]. Major risk factors include age, obesity, joint injury, abnormal mechanical loading, female sex, and genetic predisposition [3].

Current non-surgical treatments—including oral analgesics, corticosteroids, and hyaluronic acid (HA) injections—primarily alleviate symptoms but fail to halt disease progression or promote cartilage regeneration [4]. Consequently, regenerative approaches such as platelet-rich plasma (PRP) have gained interest for their potential to modulate inflammation, enhance repair, and delay surgical intervention [5]. PRP, an autologous blood product rich in growth factors (e.g., PDGF, TGF-β, VEGF), may attenuate cartilage breakdown by suppressing IL-1β, TNF-α, and MMPs while stimulating chondrocyte proliferation, extracellular matrix synthesis, and mesenchymal stem cell differentiation [2,6].

Clinical evidence indicates that PRP outperforms HA and corticosteroids in short-term pain relief and functional improvement, particularly in early-stage KOA (Kellgren–Lawrence grades I–III) [7,8]. However, results remain inconsistent: while some randomized controlled trials (RCTs) demonstrate benefits lasting 12–24 months, others—including the RESTORE trial—report no advantage over placebo at 12 months [9,10]. The biological plausibility of long-term efficacy from a limited PRP course therefore remains uncertain, especially in a progressive condition such as KOA. Despite numerous short- and mid-term RCTs, evidence beyond two years is scarce, and real-world longitudinal data are virtually absent.

This retrospective cohort study aimed to evaluate whether the effects of three intra-articular PRP injections administered over one month could persist in early-stage KOA over a 5-year follow-up. Fifty-eight patients treated with PRP were compared with 58 matched controls receiving conventional therapy (NSAIDs or HA). By employing a standardized PRP preparation protocol and a uniform multimodal conservative program, this study provides one of the longest real-world follow-ups directly comparing PRP and standardized conservative management. The findings are expected to inform clinical decision-making and protocol optimization. We hypothesized that PRP injections, as prepared and administered in this protocol, would not demonstrate sustained superiority over standardized conservative management at 5 years.

## Methods

### Ethical approval

This retrospective study was approved by the Institutional Review Board of Chongqing General Hospital (Approval No. 2023−158; approved on October 26, 2023). Written informed consent was obtained from all participants prior to data collection. The study was conducted in accordance with the Declaration of Helsinki (Fortaleza revision, 2013). All patient data were anonymized prior to analysis.

### Data access

Electronic medical records were accessed on 15 January 2025 to retrieve data for patients treated between 1 January and 31 December 2019. All identifiable information was removed prior to analysis.

### Participants and Design

This retrospective cohort study initially identified 136 consecutive patients with bilateral early-stage knee osteoarthritis (Kellgren–Lawrence grade I–II) who received either a standardized management protocol for osteoarthritis (SMPO) combined with platelet-rich plasma (PRP) injections at weeks 0, 2, and 4 (Group A), or SMPO alone (Group B), at Chongqing General Hospital between January and December 2019. Treatment assignment was not randomized; instead, it reflected real-world clinical practice where patients and physicians jointly decided on the treatment approach based on individual circumstances, including patient preference, affordability, and perceived clinical need. As a result, while baseline characteristics were comparable, the possibility of selection bias due to unmeasured confounders cannot be entirely excluded. The authors had no access to identifiable patient information during or after data collection. Because both knees in each patient received the same intervention, analyses were conducted at the patient level to avoid within-subject dependence bias. All patients had bilateral knee involvement with comparable symptom severity at baseline, defined as the same Kellgren–Lawrence grade in both knees and no clinically meaningful difference in baseline pain intensity between knees. Outcomes were analyzed at the patient level by averaging scores from both knees for all measures (VAS, WOMAC subscales, and SF-36 domains), thereby avoiding within-subject dependence. Patients with clinically significant asymmetry were not included in the final analysis.

*Standardized Management Protocol for Osteoarthritis (SMPO):*

The standardized management protocol (SMPO) for osteoarthritis consisted of a structured combination of non-pharmacological and pharmacological measures:

1. Core non-pharmacological interventions: All patients received individualized education focused on disease understanding, joint protection, and long-term lifestyle modification.
2. Weight management: A structured weight-reduction plan was implemented, targeting at least a 5% reduction in body weight in accordance with the European Society for Clinical and Economic Aspects of Osteoporosis and Osteoarthritis (ESCEO) guidelines.
3. Exercise therapy: Patients were instructed to perform daily neuromuscular training and strengthening exercises for approximately 30 minutes per day.
4. Pharmacological protocol (acute phase): For patients with refractory pain (VAS ≥ 4) at enrollment, a 2-month therapeutic course was uniformly prescribed to all patients in both groups who met this criterion, including oral nonsteroidal anti-inflammatory drugs (NSAIDs; e.g., celecoxib 200 mg once daily), topical NSAIDs (diclofenac gel three times daily), glucosamine sulfate (1500 mg daily), and diacerein (50 mg twice daily).
5. Post-treatment maintenance: After this 2-month pharmacotherapy phase, patients discontinued the active pharmacological regimen and continued only with the foundational triad of education, weight control, and regular exercise as maintenance therapy. Any additional pharmacological interventions during the 5-year follow-up period were at the discretion of treating physicians and were not systematically recorded due to the retrospective design.

*PRP Preparation:*

Peripheral venous blood (40 mL) was drawn from each patient, of which a portion was used for routine laboratory quality control, and the remaining 30 mL was processed manually in sodium citrate tubes using ACD-A as an anticoagulant, without a commercial preparation kit. A double-spin protocol was applied, consisting of a first centrifugation at 400 g for 10 minutes to separate plasma and a second centrifugation at 800 g for 15 minutes to isolate the platelet pellet. The pellet was then resuspended to a final volume of 6 mL (3 mL per knee), yielding leukocyte-poor PRP. The mean baseline platelet count was approximately 210 × 10⁹/L, while the PRP contained about 800 × 10⁹/L platelets, representing an approximately fourfold enrichment over whole blood. Marked leukocyte and erythrocyte reduction was achieved through the double-spin protocol, consistent with a leukocyte-poor PRP profile. Routine hematological analysis was performed at the time of PRP preparation in 2019 to quantify platelet, leukocyte, and erythrocyte concentrations in both whole blood and final PRP samples. Cellular composition data were derived from laboratory quality-control records archived contemporaneously with clinical preparation and were retrospectively extracted for the present analysis. PRP preparation and reporting were conducted in accordance with the Minimum Information for Studies Evaluating Biologics in Orthopaedics (MIBO) guidelines. The detailed cellular composition of leukocyte-poor PRP is summarized in Table S2 in S1 File. Routine quality control confirmed that all PRP preparations met predefined acceptance criteria (platelet count ≥500 × 10⁹/L, leukocyte count <0.5 × 10⁹/L). The mean platelet enrichment of approximately fourfold (range 2.8–5.2, coefficient of variation 15.0%) is at the lower end of the therapeutic range reported in the literature (typically 3- to 8-fold). However, previous studies have demonstrated clinical efficacy with similar enrichment levels [6,11], and no patients fell below the commonly accepted minimum threshold of 2.5-fold enrichment. The double-spin protocol resulted in a leukocyte concentration of 0.4 ± 0.2 × 10⁹/L in the final PRP (representing a > 94% reduction compared to whole blood) and an erythrocyte concentration of 0.06 ± 0.03 × 10¹²/L (>98% reduction), confirming the leukocyte-poor and erythrocyte-depleted nature of the PRP product. Detailed cellular composition across patients is presented in Table S2 in S1 File, including ranges and coefficients of variation.

*Inclusion Criteria:*

Participants were eligible for inclusion if they met all of the following criteria:

1. Radiologically confirmed bilateral knee osteoarthritis (Kellgren–Lawrence grade I–II) as determined by magnetic resonance imaging (MRI) and/or standard radiography.
2. Failure of non-operative management for ≥3 months, defined as persistent symptomatic pain with a visual analog scale (VAS) score ≥4 despite adherence to the standardized management protocol for osteoarthritis (SMPO).
3. Presence of acute-phase bilateral knee pain, with a VAS score ≥ 4 at the time of enrollment..

*Exclusion Criteria:*

Participants were excluded if they met any of the following conditions:

1. Presence of systemic inflammatory or autoimmune arthritis (e.g., rheumatoid arthritis, lupus.
2. History of major knee trauma or previous knee surgery.
3. Severe meniscal pathology associated with mechanical symptoms such as locking or catching.
4. Intra-articular injection or surgical intervention within the preceding 3 months.
5. Presence of significant hematologic, oncologic, or metabolic comorbidities.
6. Ongoing anticoagulant or antiplatelet therapy at the time of enrollment.
7. Inability or unwillingness to complete the 5-year follow-up protocol.

*Exclusion Rationale:*

A total of 136 patients with early-stage knee osteoarthritis were initially screened between 2017 and 2019. Twenty patients were excluded: eight did not meet the inclusion criteria, seven had incomplete follow-up data, and five underwent total knee arthroplasty during the follow-up period. Consequently, 116 patients were included in the final analysis, comprising 58 in the PRP group and 58 in the SMPO group (Table 1).

**Table 1:** Patient demographics.

	Group A (n = 58)	Group B (n = 58)	Statistical Value	*P Value*
Gender				
Female (%)	42（72.4）	40（69.0）	χ^2^ = 0.683	0.166
Male (%)	16（27.6）	18（31.0）		
Age (years)	50.48 ± 2.74	51.05 ± 2.74	*t* = −1.117	0.575
K-L Classification				
Grade I	32	34	χ^2^ = 0.708	0.141
Grade II	26	24		
Body Mass Index (kg/m²)	25.40 ± 0.62	25. 25 ± 0.44	*t* = 1.442	0.152

*Adherence and follow-up monitoring:*

Adherence to the prescribed exercise therapy and weight management protocols was not formally monitored during the 5-year follow-up period, as this was a retrospective study based on medical record review. Similarly, any treatment modifications, including repeat PRP injections, additional NSAID use, or surgical interventions occurring after the initial treatment phase, were not systematically documented. These limitations are acknowledged in the Discussion section.

### Observation indicators

At 5 years after the initial treatment, both patient groups underwent comprehensive evaluations using multiple standardized outcome measures. These included the acute-phase Visual Analog Scale (VAS) for pain intensity, the Western Ontario and McMaster Universities Osteoarthritis Index (WOMAC) [12] for assessing pain, stiffness, and physical function, and the SF-36 Health Survey for overall quality of life [13]. The VAS provides a simple and validated method for quantifying pain on a 0–10 scale [14]. The WOMAC index, widely used in osteoarthritis research, is a reliable instrument for evaluating the multidimensional impact of joint degeneration on daily function [15]. The SF-36 questionnaire allows a holistic assessment of health-related quality of life across multiple domains [16]. Together, these tools provide a comprehensive and multidimensional evaluation of the long-term clinical efficacy of the treatment protocols. All abbreviations used in this manuscript are listed in Table S1 in S1 File.

### Statistical analysis

Continuous variables were summarized as mean ± standard deviation (SD), and categorical variables as frequencies and percentages. Within-group comparisons between baseline and 5-year follow-up values were conducted using paired *t*-tests for normally distributed variables, while between-group differences were evaluated using independent t-tests or χ² tests, as appropriate. Because only two time points (baseline and 5-year follow-up) were available and no intermediate data were collected, repeated-measures or longitudinal models (e.g., mixed-effects analysis) were not applicable. Multivariable regression analysis was not performed due to the limited sample size and incomplete covariate data for some participants (e.g., symptom duration, activity level, occupational factors, adherence to exercise and medication). Accordingly, the present analyses represent unadjusted comparisons describing observed long-term trends rather than establishing causal relationships. Because the primary endpoint was the between-group difference in VAS at 5 years, no formal multiplicity correction was applied. Secondary outcomes should be interpreted as exploratory. The analyses should be interpreted as exploratory. All statistical analyses were conducted using SPSS version 26.0 (IBM Corp., Armonk, NY, USA), with a two-tailed *P* value <0.05 considered statistically significant. All data entries and analyses were independently verified by two investigators, and any discrepancies were resolved by consensus prior to final analysis.

## Results

### Patient selection and baseline characteristics

**Fig 1:**
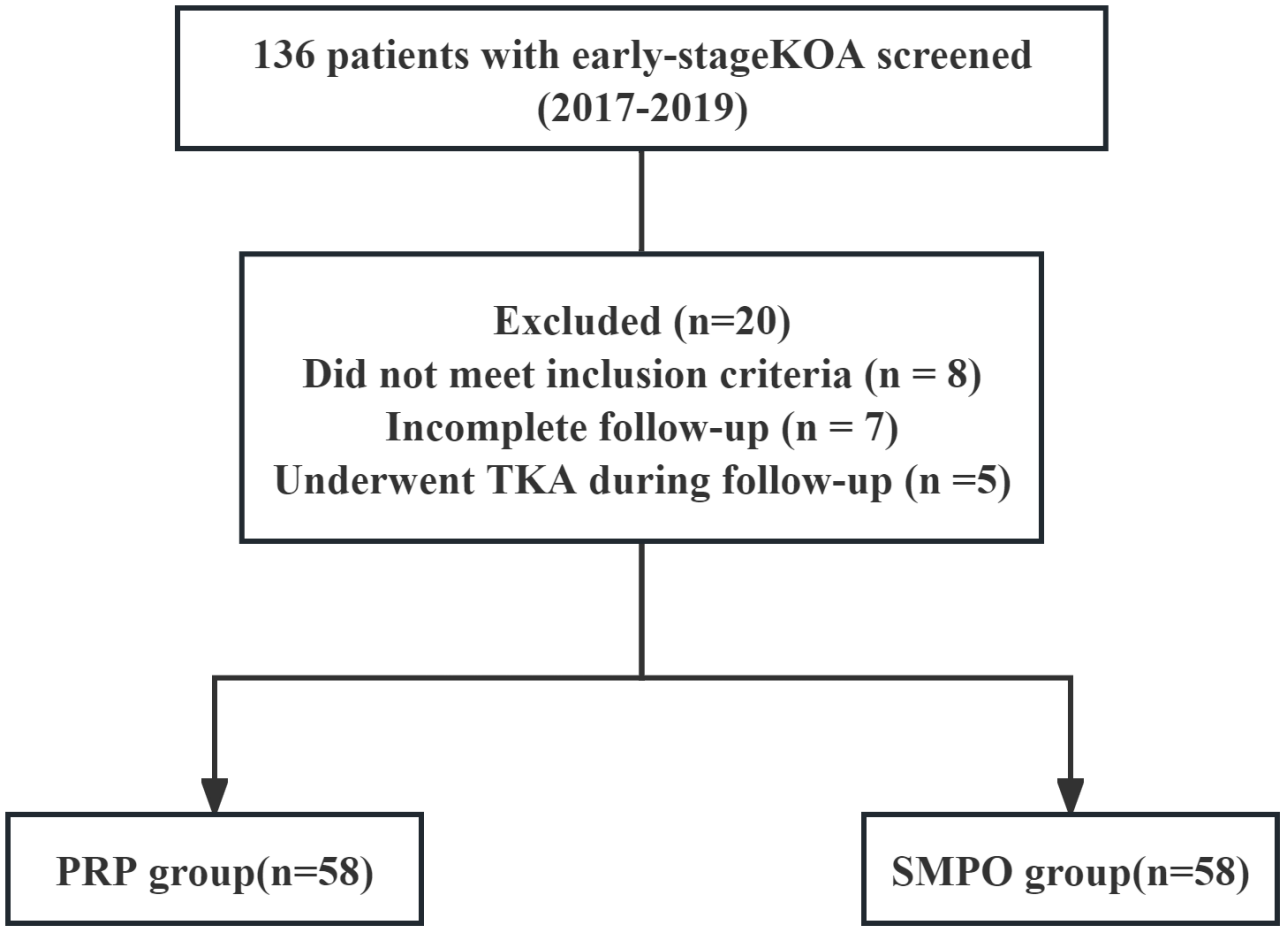
Flow diagram of patient selection and inclusion process.

Between January and December 2019, a total of 136 patients with early-stage knee osteoarthritis (Kellgren–Lawrence grade I–II) were screened for eligibility at Chongqing General Hospital. After excluding eight patients who did not meet the inclusion criteria, seven with incomplete follow-up data, and five who underwent total knee arthroplasty during the follow-up period, 116 patients were included in the final analysis—58 who received standardized management combined with platelet-rich plasma (PRP) injections (Group A) and 58 who received standardized management alone (Group B). Baseline demographic and clinical characteristics, including age, sex, body mass index (BMI), affected side, Kellgren–Lawrence grade, and baseline VAS and WOMAC scores, were comparable between the two groups (all P > 0.05), indicating good baseline balance (Table 1). The patient screening and selection process is summarized in Fig 1. All 116 included patients had symmetric bilateral involvement at baseline as defined in the Methods, and no patients required exclusion or separate analysis due to asymmetric severity.

### Missing data

Analyses were conducted on complete cases. The proportion of missing values for any variable was below 5%. Missing data were distributed as follows: 3 patients (2 in Group A, 1 in Group B) had incomplete SF-36 domain scores, and 2 patients (1 per group) had missing WOMAC stiffness scores at 5 years. No systematic pattern of missingness was identified, and given the low proportion, the potential for bias is considered minimal.

Data are presented as mean ± SD or n (%). Analyses were based on complete cases. Missing values per variable were <5%.

### Visual analog scale

The study compared baseline and 5-year Visual Analog Scale (VAS) pain scores between Group A (n = 58) and Group B (n = 58). At baseline, the mean VAS score was 4.64 ± 0.52 in Group A and 4.80 ± 0.59 in Group B, with no statistically significant difference (*t* = −1.510, *P* = 0.406). After 5 years, mean VAS scores increased in both groups — to 5.41 ± 1.17 in Group A and 5.44 ± 1.16 in Group B — yet the difference between groups remained non-significant (*t* = −0.160, *P* = 0.849). These results indicate no long-term divergence in pain outcomes between the two treatment arms over the 5-year follow-up period (Table 2).

**Table 2:** Visual analog scale (VAS) pain scores at baseline and 5-year follow-up in Group A (SMPO + PRP) and Group B (SMPO only).

	Group A (n = 58)	Group B (n = 58)	Statistical Value	*P Value*
VAS score				
Baseline	4.64 ± 0.52	4.80 ± 0.59	*t* = −1.510	0.406
5^th^ year	5.41 ± 1.17	5.44 ± 1.16	*t* = −0.160	0.849

### WOMAC score

WOMAC subscale scores for pain, stiffness, and physical function were compared between Group A (n = 58) and Group B (n = 58) at baseline and at the 5-year follow-up. At baseline, there were no significant differences between groups (pain: 8.41 ± 2.12 vs. 8.64 ± 2.35, *t* = −0.620, *P* = 0.537). After 5 years, WOMAC scores worsened in both groups—for example, pain increased to 9.76 ± 3.52 in Group A and 9.43 ± 3.41 in Group B (*t* = 0.520, *P* = 0.606). Similar patterns were observed for stiffness and physical function. No statistically significant between-group differences were found at either time point (*P* > 0.05 for all comparisons; Table 3), indicating parallel declines in WOMAC outcomes over the 5-year period. Although mean VAS and WOMAC scores deteriorated in both groups, the between-group differences remained minimal and clinically negligible.

**Table 3:** WOMAC subscale scores (pain, stiffness, function) at baseline and 5-year follow-up.

	Group A (n = 58)	Group B (n = 58)	Statistical Value	*P Value*
Pain				
Baseline	8.41 ± 2.12	8.64 ± 2.35	*t* = −0.620	0.537
5^th^ year	9.76 ± 3.52	9.43 ± 3.41	*t* = 0.520	0.606
Stiffness				
Baseline	3.02 ± 0.82	2.98 ± 0.85	*t* = −0.240	0.812
5^th^ year	4.02 ± 1.48	3.95 ± 1.52	*t* = 0.250	0.801
Physical Function				
Baseline	19.83 ± 3.21	20.12 ± 3.07	*t* = −0.480	0.634
5^th^ year	26.53 ± 7.89	25.78 ± 8.12	*t* = 0.480	0.632

### SF-36 health survey

SF-36 Health Survey scores were compared between Group A (n = 58) and Group B (n = 58) at baseline and at the 5-year follow-up. Across all eight domains (e.g., Physical Functioning, Role Physical, Bodily Pain), baseline scores showed no significant differences between groups (*P* > 0.05). After 5 years, scores declined comparably in both groups across most domains. For example, Physical Functioning decreased from 57.34 ± 10.89 (Group A) and 57.12 ± 11.25 (Group B) at baseline to 49.78 ± 6.89 and 49.64 ± 6.52, respectively (*t* = 0.110–1.210, *P* = 0.230–0.920). Similarly, Role Emotional scores declined from 74.16 ± 5.62 (Group A) and 75.05 ± 5.94 (Group B) to 68.24 ± 6.37 and 69.16 ± 6.72 (*t* = 0.820, *P* = 0.410). No statistically significant between-group differences were observed at either time point for any SF-36 domain (*P* > 0.05 for all comparisons; Table 4). These findings indicate parallel declines in health-related quality of life over 5 years in both treatment arms, with no meaningful intergroup differences.

**Table 4:** SF-36 health survey.

	Group A (n = 58)	Group B (n = 58)	Statistical Value	*P Value*
Physical Functioning				
Baseline	57.34 ± 10.89	57.12 ± 11.25	*t* = 0.110	0.910
5^th^ year	49.78 ± 6.89	49.64 ± 6.52	*t* = 0.130	0.900
Role Physical				
Baseline	53.62 ± 7.85	53.48 ± 8.12	*t* = 0.100	0.920
5^th^ year	47.12 ± 4.87	48.05 ± 5.12	*t* = 1.210	0.230
Bodily Pain				
Baseline	54.08 ± 4.95	54.97 ± 5.21	*t* = 1.020	0.310
5^th^ year	47.26 ± 5.32	48.14 ± 5.67	*t* = 0.940	0.350
General Health				
Baseline	61.34 ± 5.72	62.95 ± 6.08	*t* = 1.520	0.130
5^th^ year	55.24 ± 6.52	56.12 ± 6.81	*t* = 0.760	0.450
Vitality				
Baseline	65.34 ± 5.67	67.12 ± 5.82	*t* = 1.640	0.100
5^th^ year	62.14 ± 6.37	63.26 ± 6.72	*t* = 0.980	0.330
Social Functioning				
Baseline	70.53 ± 6.21	71.63 ± 6.58	*t* = 1.120	0.260
5^th^ year	64.52 ± 5.47	65.43 ± 5.82	*t* = 0.910	0.360
Role Emotional				
Baseline	74.16 ± 5.62	75.05 ± 5.94	*t* = 0.890	0.380
5^th^ year	68.24 ± 6.37	69.16 ± 6.72	*t* = 0.820	0.410
Mental Health				
Baseline	76.84 ± 4.65	78.24 ± 5.12	*t* = 1.350	0.180
5^th^ year	67.84 ± 6.82	69.05 ± 7.15	*t* = 1.280	0.200
Health Change				
Baseline	3.02 ± 0.29	3.10 ± 0.31	*t* = 0.810	0.420
5^th^ year	3.37 ± 0.32	3.48 ± 0.35	*t* = 0.890	0.380

### Intra-group comparisons

Within-group analyses from baseline to the 5-year follow-up revealed significant deterioration in both treatment groups. In Group A (PRP + SMPO), VAS scores increased from 4.64 ± 0.52 to 5.41 ± 1.17 (*P* < 0.001), total WOMAC scores worsened from 37.1 ± 9.5 to 51.9 ± 11.7 (*P* < 0.001), and the SF-36 physical component summary (PCS) score declined from 56.2 ± 11.2 to 48.6 ± 9.4 (*P* = 0.002). Similarly, in Group B (SMPO only), VAS increased from 4.80 ± 0.59 to 5.44 ± 1.16 (*P* < 0.001), WOMAC from 36.4 ± 10.3 to 52.6 ± 12.1 (*P* < 0.001), and SF-36 PCS from 55.9 ± 10.8 to 47.8 ± 10.1 (*P* = 0.001). These results indicate that both groups experienced significant declines in pain, function, and quality of life over time, consistent with the natural progression of knee osteoarthritis. However, no statistically significant between-group differences were observed at the 5-year endpoint for any of these outcome measures (*P* > 0.05).

These findings suggest that the symptomatic benefits of a single PRP course may not be sufficient to alter the natural progression of early KOA.

## Discussion

To our knowledge, this study represents one of the longest real-world follow-ups evaluating the long-term efficacy of PRP in early-stage KOA, providing valuable evidence on the temporal trajectory of symptom changes after a short-course PRP regimen. Our 5-year follow-up findings indicate that intra-articular platelet-rich plasma (PRP) injections, as prepared and administered in this protocol, did not confer statistically significant benefits over standardized conservative management in terms of pain, function, or quality of life. These results align with several recent randomized controlled trials and systematic reviews that have questioned the durability of PRP’s clinical effects beyond the short-term improvements observed in previous studies [5,9,10,17,18].

The absence of long-term efficacy may be mechanistically linked to the transient lifespan of growth factors within PRP (typically <7 days) and the lack of sustained modulation of the inflammatory microenvironment [19]. Without repeated biological stimulation, catabolic processes driven by IL-1β and TNF-α likely resume, leading to progressive matrix degradation [20]. Although PRP can transiently modulate inflammatory mediators, promote chondrocyte proliferation, and enhance extracellular matrix synthesis [2,6], our results and prior longitudinal studies indicate that these benefits wane over time [9–11,21]. This may reflect the progressive and multifactorial nature of osteoarthritis, in which transient biological modulation is insufficient to alter long-term disease trajectory [2,4,22].

Variability in PRP preparation, platelet concentration, cytokine profile, and injection protocols contributes to inconsistent clinical outcomes [5,6,11,22]. Even with a standardized leukocyte-poor protocol, our results were consistent with meta-analytic data showing that PRP’s therapeutic advantage diminishes with time [23].

Beyond randomized controlled trials, accumulating real-world evidence (RWE) has provided important insights into the long-term effectiveness of PRP for knee osteoarthritis. Recent observational and registry-based studies have reported that although PRP may be associated with short-term symptom relief, its clinical benefits tend to diminish over time, with no clear superiority over standard conservative treatments beyond mid-term follow-up. For example, a real-world cohort study demonstrated comparable long-term outcomes between PRP and conventional non-surgical management in patients with knee osteoarthritis [24]. Similarly, real-world data from routine clinical practice failed to show sustained functional advantages of PRP compared with established conservative therapies [25]. Our findings are consistent with these real-world observations and further extend the existing evidence by providing a five-year comparative follow-up under standardized PRP preparation and conservative management protocols.

Both the PRP and control groups—managed with standardized conservative therapy—exhibited parallel declines in function and quality of life, indicating that conventional management remains equally effective in the long term. Similar findings have been reported in comparative studies and systematic reviews involving hyaluronic acid and corticosteroid injections [8,26].

Clinically, these findings highlight the need to manage patient expectations regarding PRP therapy for KOA. While transient improvements may occur, sustained pain relief and functional benefits were not demonstrated at 5 years. PRP should thus be viewed as an adjunct rather than a definitive long-term treatment, particularly given its cost and patient burden [2,8,17,27].

An additional consideration in interpreting the long-term outcomes is the potential interaction between PRP therapy and concomitant nonsteroidal anti-inflammatory drug (NSAID) use. NSAIDs exert their analgesic and anti-inflammatory effects primarily through inhibition of cyclooxygenase (COX) activity, which may interfere with platelet activation and degranulation. Experimental studies have shown that NSAID exposure can reduce the release of platelet-derived growth factors, including platelet-derived growth factor (PDGF) and transforming growth factor-β (TGF-β), potentially attenuating the biological activity of PRP. In real-world clinical practice, PRP injections are frequently administered alongside NSAIDs for symptom control, and such pharmacological interference may partially neutralize the intended regenerative and anti-inflammatory effects of PRP. This interaction may partly explain the lack of sustained long-term superiority of PRP observed in real-world settings.

Beyond statistical significance, the clinical relevance of our findings warrants consideration. The between-group differences at 5 years were minimal: VAS −0.03 (95% CI −0.46 to 0.40), WOMAC pain 0.33 (95% CI −0.94 to 1.60), and WOMAC function 0.75 (95% CI −2.19 to 3.69). For VAS, the confidence interval lies entirely below the established minimal clinically important difference (MCID) of 1.0 [14], indicating that any true difference is unlikely to be clinically meaningful. For WOMAC pain and function, however, the confidence intervals are wide and include the respective MCID thresholds (1.5 and 3.0) [15], reflecting the substantial inter-patient variability observed. This suggests that while the average treatment effect is negligible, individual responses may vary, and we cannot entirely rule out the possibility of clinically meaningful benefits in some patients.

The relatively large standard deviations observed (e.g., VAS SD ~ 1.2) indicate substantial inter-patient variability in outcomes. This heterogeneity may reflect differences in baseline disease severity, inflammatory status, or individual response to treatment. While we were unable to perform reliable subgroup analyses due to the modest sample size, this variability suggests that certain patient subsets may derive greater benefit from PRP than others. Future studies with larger cohorts should explore potential predictors of response, such as age, BMI, baseline inflammatory markers, or synovitis severity on imaging.

This study has several limitations, including its retrospective, single-center design, lack of intermediate imaging and biomarker data, modest sample size, and absence of PRP compositional analysis. Adherence to exercise, weight management, and medication was not formally monitored during follow-up, which may have influenced outcomes. Additionally, we did not systematically record any subsequent treatments—such as repeat PRP injections, additional NSAID use, or surgical interventions—that may have occurred during the 5-year period. In particular, long-term NSAID usage patterns, which could interact with PRP efficacy as discussed earlier, were not captured. This represents an important gap in our data and may confound the interpretation of the null findings. Additionally, we did not adjust for multiple comparisons, which increases the risk of Type I error; however, the consistent non-significance across all between-group comparisons supports the robustness of the findings. An additional limitation is the lack of intermediate outcome assessments between baseline and the 5-year follow-up. With only two time points, we are unable to characterize the temporal trajectory of treatment effects. Specifically, it is impossible to determine whether PRP provided short-term benefits that subsequently waned, whether there was never any treatment effect, or whether both groups improved initially before returning to baseline trajectory. This is particularly relevant given that previous randomized controlled trials have reported symptomatic benefits of PRP at 6–12 months [21,9]. Our study design cannot confirm or refute these short- to mid-term findings, and the absence of intermediate data limits our ability to draw conclusions about the duration of any potential PRP effect. Future prospective studies should include serial assessments at multiple time points to capture the dynamic course of treatment response.

The retrospective, non-randomized design introduces the possibility of selection bias and residual confounding despite comparable baseline characteristics. Factors such as patient motivation, socioeconomic background, and activity level were not captured and could have influenced both treatment choice and long-term outcomes. Therefore, our findings should be interpreted as hypothesis-generating rather than confirmatory. Future prospective randomized trials with comprehensive covariate collection and systematic monitoring of adherence and co-interventions are warranted to validate these observations. Nevertheless, the study provides valuable real-world longitudinal data under standardized preparation and treatment conditions.

Future investigations should include multicenter, randomized controlled trials directly comparing leukocyte-rich versus leukocyte-poor PRP, assessing repeated versus single dosing regimens, and integrating imaging and biomarker endpoints to elucidate mechanisms of response [28,29]. Identifying responder phenotypes based on inflammatory or metabolic biomarkers may also facilitate personalized treatment strategies. Additionally, rigorous cost-effectiveness analyses are warranted to evaluate long-term clinical and economic value.

In summary, a short course of leukocyte-poor PRP did not confer sustained benefit compared with standardized conservative management over five years. In conclusion, these findings delineate the temporal boundaries of PRP efficacy and emphasize the need for evidence-based, patient-tailored application rather than routine use in early-stage knee osteoarthritis, thereby guiding more rational and personalized integration of PRP within future conservative management strategies.

## Supporting information

S1 Data De-identified individual-level clinical data (baseline and 5-year follow-up) for all outcome measures. This file contains 26 Excel spreadsheets corresponding to VAS, WOMAC subscales, and SF-36 domains.
(RAR)

S1 File Abbreviations used in the manuscript and cellular composition of leukocyte-poor PRP. (This file contains Table S1 and Table S2.).
(DOCX)
