# Supplementary material for: Five-year real-world outcomes of short-course leukocyte-poor PRP versus standard conservative therapy in early-stage knee osteoarthritis
Source: PLoS One. 2026 Apr 2;21(4):e0344749. doi: 10.1371/journal.pone.0344749 (PMC13046112; doi:10.1371/journal.pone.0344749)
Supplement: S1 File — (This file contains Table S1 and Table S2.). (DOCX) [file pone.0344749.s002.docx]

**Table S1**. List of abbreviations used in the manuscript

| Abbreviation | Full term |
| --- | --- |
| KOA | Knee Osteoarthritis |
| PRP | Platelet-Rich Plasma |
| LP-PRP | Leukocyte-Poor Platelet-Rich Plasma |
| SMPO | Standardized Management Protocol for Osteoarthritis |
| VAS | Visual Analog Scale |
| WOMAC | Western Ontario and McMaster Universities Osteoarthritis Index |
| SF-36 | Short Form-36 Health Survey |
| NSAIDs | Nonsteroidal Anti-inflammatory Drugs |
| HA | Hyaluronic Acid |
| BMI | Body Mass Index |
| IRB | Institutional Review Board |
| K-L | Kellgren–Lawrence |

**Table S2**. Cellular composition of whole blood and leukocyte-poor PRP

| Parameter | Whole blood (mean ± SD) | PRP (mean ± SD) | Range (PRP) | Fold change | Coefficient of variation (PRP) |
| --- | --- | --- | --- | --- | --- |
| Platelet count (×10⁹/L) | 210 ± 35 | 800 ± 120 | 580–1090 | 3.8 | 15.0% |
| Leukocyte count (×10⁹/L) | 6.5 ± 1.8 | 0.4 ± 0.2 | 0.1–0.8 | 0.06 | 50.0% |
| Erythrocyte count (×10¹²/L) | 4.6 ± 0.5 | <0.1 | 0.02–0.12 | 0.013 | 50.0% |

Footnote: All PRP preparations met predefined acceptance criteria (platelet count ≥500 × 10⁹/L, leukocyte count <1.0 × 10⁹/L). Platelet enrichment ranged from 2.8- to 5.2-fold across patients, with no preparations falling below the minimum therapeutic threshold of 2.5-fold enrichment. The double-spin protocol achieved >94% leukocyte reduction and >98% erythrocyte reduction compared to whole blood, confirming the leukocyte-poor, erythrocyte-depleted nature of the PRP product.
